# Health care of the disadvantaged: chronic obstructive pulmonary disease in later life

**DOI:** 10.3389/fpubh.2023.1304494

**Published:** 2023-11-09

**Authors:** Fredrik Nyman

**Affiliations:** Department of Psychology and Social Work, Mid Sweden University, Östersund, Sweden

**Keywords:** United Kingdom, breathlessness, chronic disease, ethnography, respiratory disease, stigma, support groups

## Abstract

**Introduction:**

Chronic diseases have emerged as the foremost causes of death and disability worldwide. This article employs an ethnographic approach to conduct a gerontological investigation of chronic obstructive pulmonary disease (COPD), the third leading cause of global mortality, trailing only cardiovascular diseases and cancers.

**Methods:**

This study is rooted in an extensive amalgamation of biomedical literature and official epidemiological data. Additionally, it offers enriched insights through an extensive ethnographic research methodology, encompassing ethnographic fieldwork, participant observation, interviews, and focus groups.

**Results:**

The findings expound that individuals grappling with chronic obstructive pulmonary disease often undergo intricate cognitive and emotional experiences, necessitating holistic solutions that consider psychological processes, contextual factors, and subjective age. These challenges extend beyond the purview of a purely medical perspective.

**Conclusion:**

This article concludes that the lens of gerontology is invaluable in comprehending chronic obstructive pulmonary disease, particularly due to its association with old age and increased longevity. Among older individuals, diagnosing the condition presents a formidable challenge. Breathlessness, a cardinal symptom, often overlaps with normal age-related declines in pulmonary function, rendering COPD’s insidious onset misconstrued as a consequence of aging-related changes.

## 1. Introduction

More people than ever before grow to ‘extreme old age’ and communities across the globe are now facing a situation without precedent: soon there will be more people over 65 than under 18 (1). These age terms are in quotation marks because old age is not easy to define; whereas 18–29-year-olds on average consider the start of old age to be 60, people over 65 on average consider it to be 74 (2). This makes old age a subjective and age-related experience. It is, however, a fact that, with improvements in sanitation, housing, and education throughout the nineteenth and early twentieth centuries, there has been a steady decline in early and mid-life mortality (3, 4). As a consequence, life expectancy over the past century has increased by around 3 years every decade (5). In the industrialized world Hong Kong lands on top with an estimated 85.29 years [i.e., 88.17 for women and 82.38 for men (6)]. Moreover, these are mere abstractions and some national statistics have shown that individual women and men alike may survive to surpass these numbers (7, 8). In 2018, the United Nations proclaimed that there were 499,198 centenarians around the world (9). In reaction to this increasing life expectancy, the World Health Organization (10) has declared that ageing well ‘must be a global priority’ (p. 265).

The reason behind this initiative is that with increasing life expectancy, there is now a large group of older persons with increased health risks; in particular chronic diseases. Chronic diseases are now the leading causes of death and disability worldwide (11). From a neoliberal economic perspective, this group represents a so-called ‘major financial burden’ (12–14); whereas from a humanist perspective, this is a group that should be understood as potentially disadvantaged people who often experience a lower quality of life, with both physical and psychological reduced well-being (15, 16). This makes the science of gerontology now more relevant than ever (17). Gerontology is understood as the study of the social, cultural, psychological, cognitive, physical, and biological aspects and implications of ageing and persons in later life. The coinage of the term is often ascribed to Nobel Prize winner (and the ‘father of innate immunity’) Ilya Ilyich Mechnikov, much due to his emerging study of ageing and longevity while at the Pasteur Institute in Paris (18).

## 2. Materials and methods

This article employs an ethnographic case study approach to conduct a gerontological investigation of chronic obstructive pulmonary disease (COPD), which is the third leading cause of death worldwide after cardiovascular diseases and cancers (16). Understanding the impact of COPD on a person’s daily life, including their physical, psychological, and social well-being, is essential. Case studies highlight the real-world implications of COPD and can help improve patient quality of life through targeted interventions and support. As such, studying COPD through a case study approach is instrumental in advancing our knowledge, improving patient care, and addressing the multifaceted challenges associated with this debilitating respiratory condition. COPD is often misdiagnosed or undiagnosed due to its subtle and variable symptoms (19). Case studies can shed light on the challenges of accurate diagnosis, leading to improved early detection and more timely intervention. As will be explained and deliberated throughout this article, COPD is a particularly important disease to study from a holistic, gerontological viewpoint (20, 21). The specifics of COPD and how they often lead to misdiagnosis, late recognition, and stigma will be discussed in detail. The article then also provides a discussion of the potential benefits and opportunities that social support groups have to offer older persons living with a chronic disease, as well as their limitations and disadvantages. In the conclusion, the implications this has for health policy specifically for COPD, and more generally for chronic disease among older persons, is discussed and explained.

This article draws its foundation from a comprehensive synthesis of biomedical literature and official epidemiological statistics [e.g., BLF (15, 22), Raleigh (8), UNECE (23), and WHO (1, 10, 16)], in order to comprehensively analyze the epidemiological landscape of COPD on a global and local scale. Additionally, it offers enriched insights through an extensive ethnographic research exploration conducted in the United Kingdom. Between April and October 2017, as well as from January to August 2018, the ethnographic study in question (20) delved into the experiences and daily lives of older adults coping with COPD. The overall approach encompassed ethnographic fieldwork, participant observation, 30 interviews (with patients and their families, health professionals, and health policy-makers), and focus groups, collectively providing a comprehensive understanding of people’s lives with COPD. In particular, monthly meetings of three so-called ‘Breathe Easy’ groups (social support groups of the BLF) were attended (where participant observation took place and also where focus groups were conducted). These provided a further opportunity to get in touch with COPD patients, their families, and other people who were part of their daily lives, such as caregivers and volunteers. Home visits were made, as well as attending various social activities (i.e., fieldtrips, outdoor walks) in different places around the United Kingdom. The study’s full methodology is described in detail elsewhere [see Nyman (20)]. Furthermore, being that COPD is a respiratory disease, the topic of this article is a timely contribution apropos the coronavirus pandemic (COVID-19). As such, comparisons will be made between the two diseases. That said, the author would like to make explicit that the study from which this article stems was conducted in the pre-pandemic period and thus, only second-hand data will be used in making these comparisons.

For transparency purposes with regards to terminology, as illustrated, this article principally employs the term ‘patient’—rather than, e.g., client, service-user, sufferer, or survivor. While far from uncomplicated, as the word ‘patient’ does conjure up a vision of both quiet suffering and of an unequal relationship between the user of healthcare services and the provider (24), this article primarily looks to Simmons and colleagues (25) who illustrate in their study that ‘patient’ is, indeed, the most preferred term in consultations with healthcare services. As their data shows, 72.6% of participants preferred the term ‘patient’ before any other; by comparison, the word ‘client’ was liked by 43.1% [(25), p. 22]. The word ‘service-user’ was disliked more than liked overall, and only a significant minority of participants wished to be regarded as a ‘survivor’ or ‘user’ (25). In addition, as to avoid labeling people, care and research charities around the United Kingdom generally suggest using ‘positive language’—i.e., terms that focus on the individual person, rather than on the medical condition or behavior. For this reason, ‘sufferer’ is seldomly used amongst health professionals or patient organizations and their members [e.g., (26, 27)]. Lastly, empirically speaking, the large majority of the interviewees cited in this article solely referred to themselves as ‘patients’ (which corresponds with Simmons and colleagues’ study). As such, the article chooses to adopt an evidence-based terminology in referring to people who use healthcare services.

## 3. Results

### 3.1. Adopting a gerontological lens for understanding COPD

COPD is a respiratory disease in which the airways become inflamed and the air sacs in the lungs are damaged (15, 22). The oxygen level is reduced, and thus it becomes more difficult to breathe. As a result, a person has less energy and physical ability (28). As the COPD patients in this study described, doing normal things like climbing stairs, or washing and dressing themselves can be difficult or near impossible. This can be a very negative and scary experience and adjusting to the disability often is a process that takes time. For example, one research participant explained:

If my wife says to me: “You know… that bush will have to come out of the ground” … Instinctively, I go towards the garage to get a spade and dig it out. And I’ve actually gotten to the stage where I’ve gotten to the place and realize that I cannot do it. Whereas now, a mind-set sets in, and you think “I cannot do that.”

COPD is a disease that predominantly affects older adults and is also two-and-a-half times more common among the most disadvantaged people in society, where lifestyle-related risks of COPD are more prevalent (15). While there is a genetic component, the main cause of disease is the inhalation of harmful substances (15). There is no cure for COPD and thus the traditional medical model that focuses on treating pathology remains inadequate (28). But the disease can be managed, and there are several treatments available and self-management can improve a person’s symptoms and reduce exacerbations (20, 22).

For patients, the symptoms are an important determinant of their quality of life. This applies in particular to breathlessness, which is the first vital symptom of chronic respiratory disease (29). Breathlessness is described not only as something that interferes with everyday activities, but also as something that can be very frightening, as the following research participant explains:

(…) very, very scary. Because sometimes…… You cannot breathe! So, you cannot pick up the phone to tell someone… You know, one of the services, what is wrong—there is nobody there to help you. That is the most frightening bit; being on your own when you have an attack.

Based on these findings, we can conclude that with respect to this medical condition, people experience certain cognitions and emotions that in effect require holistic answers that account for psychological processes, the context, and subjective age, rather than that they can be solved from a purely medical perspective. From the *Burden of Treatment Theory*, it can be understood that among older persons with COPD, well-being is greatly affected by the effort that it costs them to manage their health and the impact COPD has on their functioning [i.e., self-management, medical consultations, and adjusting one’s lifestyle (30)]. This is referred to as the ‘treatment burden’, which cannot be measured objectively, but rather is a subjective experience that is greatly affected by someone’s perceived social support and ‘psychological capital’. Psychological capital refers to a person’s capacity to positively respond to situations (mentally or behaviourally) and consists of self-efficacy, resilience, hope, and optimism (30). Research shows that social support increases psychological capital, and that perceived social support also directly decreases the experienced treatment burden (30). A problem is, however, that among older persons, social isolation can be a compounding factor, as people tend to get less mobile, may lose their partner or good friends (due to old age or disease) and have fewer possibilities of meeting other people—in contrast to what people at younger ages do at school, study, or work (31). In addition, COPD has some specific characteristics that hinder social support and psychological capital, as will be described in the next section.

### 3.2. COPD as an invisible and stigmatized disease

COPD can be described and understood as an invisible disease (15, 19). From the outside, you cannot see that someone is living with the disease. Moreover, the disease is often confused with complaints that arise from old age, from smoking and/or in connection with an unhealthy lifestyle that leads to physical problems, such as obesity. The first signs can therefore be misdiagnosed or not be understood by the patients themselves as something that requires medical attention. As a result, COPD is often diagnosed at a late stage and there are probably many people who have no diagnosis yet, but do have symptoms (15). What is more, following the COVID-19 outbreak in 2019–2020, symptoms of COPD may now be even more misinterpreted due to the similarities in clinical presentation. One important distinction, however, is that COVID-19 often presents itself with fever—which COPD does not (32). Needless to say, COVID-19 does cause symptoms that can be particularly severe for persons with COPD. Explicitly, persons with COPD are not at higher risk to contract the coronavirus; however, they are at higher risk for complications and worse health outcomes (33, 34). In fact, a report from the Centers for Disease Control and Prevention (CDC) in the United States suggests that over one third of adult patients hospitalized with COVID-19 were respiratory related; COPD, in particular (35). In fact, Yohannes writes that:

…the chronic nature of COPD, the severity of lung function impairment, and concomitant low-grade inflammation may compromise immune mechanisms and predispose to acquiring acute respiratory infections. Without adequate and prompt treatment, COVID-19 infection may progress to intolerable dyspnea, hypoxia, and tenacious dry cough, excessive fatigue, and with or without sputum production from the underlying COPD. Some may develop pneumonia, leading to a hospital admission, requirements for intensive care, and eventually for some, death might occur. (p. 5).

Although COPD cannot be cured, progression can be slowed down and help with symptom management can thus be important (15). It should be emphasized once again that ‘invisible’ is not to be confused with not being severe. As invisible as the disease is called, its symptoms can be noticeable and ever ‘present’, as became very evident during the ethnographic study from which this article stems (20). To illustrate this: during a conversation at two participants’ home, the person with COPD left the room to visit the bathroom. Although the conversation with the spouse continued, it was difficult not to be distracted by the coughing that was audible (even later when listening back to the interview recording). As such, persons with COPD risk social embarrassment and stigmatization in many ways. In various public and social settings, the severe breathlessness and disruptive cough can be embarrassing for the individual person. At the same time, ‘witnessing these symptoms may make bystanders uncomfortable, leading to more social awkwardness’ [(36), p. 917]. The invisibility of COPD has thus a social element, while also being both political and economic in the clinical context. Yet the stigmatization may in fact have turned for the worse following COVID-19, especially when it comes to chronic coughing. As Bouayed writes (37), sneezing and coughing.

…are important defensive reflex responses to expel noxious xenobiotics including infectious agents or irritants, and are also common symptoms of many allergies. However, they further permit the transmission of pathogens in the case of respiratory infectious diseases (p. 57).

Coughing, a symptom that is present ‘in up to 70% of symptomatic COVID-19 patients’ [(37), p. 57], remains the primary reason for COVID-19-related stigma. In fact, it has been reported that coughing has constituted a significant stigma-related factor for COVID-19 patients with cough, ‘leading to their social isolation’ (37, 38). Thus, imagine how this may affect persons who already live with a chronic cough—whether due to COPD or another respiratory disease. For instance, in their article on cough in adolescents with cystic fibrosis (CF), Taheri and colleagues (39) write how the COVID-19 pandemic has ‘significantly disrupted the lives of adolescent patients with *CF* and their parents’ (p. 2). Persons with COPD share these experiences. While cough is recognized as a common symptom in respiratory disease, ‘research has not focused on patients’ experiences of cough and any associated challenges regarding this manifestation, especially during the pandemic of COVID-19 [(40), p. 2]. Frequent coughing may cause major challenges and embarrassment for the individual—even more so during the COVID pandemic, when people were acutely sensitive and aware about this protective mechanism. Persons with COPD cannot hide their cough. Thus, aside from taking care of themselves and managing their disease, persons with COPD have also had to overcome ‘issues related to social stigma and isolation’ [(40), p. 1]. That said, while public perceptions of chronic coughing may come to change in a post COVID-19 future, for persons with COPD the experience of rejection and isolation may come to persist.

Importantly, whereas they need social support, persons with COPD usually experience a loss of social activities and social contacts in their lives due to lack of energy and physical ability, but also because it can often be unpleasant for others to observe a person (with COPD) cough and/or being short of breath (36). Moreover, if patients have difficulty adjusting to their illness they may act their frustrations out on others; this can also lead to stress, conflict and important others taking their distance instead of providing so-needed social support (41). Having close family and especially a positive partner-relationship often proves to be an enormous support, but at the same time a partner also has to deal with an enormous burden of providing care (13, 42, 43). Considering the physical impossibilities, both spouses can be seen as victims of COPD, as was mentioned by one research participant during an interview:

She said, “When I was diagnosed… we were all in complete shock.” Her family, that is. And she said, “What’s happened is that I’m supporting them, because they are so shocked… They’re all supporting me, but I’m the one that’s being strong.”

Moreover, there is another obstacle and that is that COPD is often caused by smoking (10, 15). This makes it a stigmatized disease in which both others and the patients themselves can have the feeling that they have self-inflicted the disease upon themselves (44). COPD is largely a preventable disease (15, 44). Most of the patients in this study had stopped smoking after their diagnosis, but unfortunately, they were too late; the damage to the lungs had become permanent and no longer reversible. As another research participant explained:

I know it [COPD] gets worse, so… that’s why I keep active and things. But I did smoke, and I stopped that in 2008. I had a cough… and I had to go for a ‘blow test’ [spirometry] and they showed me: “That’s where you are. And that’s where you should be.” From there on, I stopped smoking. So…… I will not be going back to it. One, I cannot afford it. And two—[laughs] I think it’s stupid! I keep telling my grandchildren: “Do not let me catch you smoking!” You know… And when I see people out on the street, I just want to go over and say: “Look. This is what happens to you.” [she points to herself].

For some, the stigma feels unjustified because at the time they started smoking they were not fully aware of the risks and smoking was still generally accepted (44). For others, as this and other studies have shown, they are ashamed and have delayed seeking help for their complaints for a long time (15). Negative reactions from others, including medical professionals, are certainly present (19).

### 3.3. Social support groups

There are several charities in the United Kingdom concerned with respiratory diseases and the people affected by them. That said, the British Lung Foundation (BLF) is the only charity concerned with all types of respiratory conditions. As one of their patient services, the BLF organizes social support groups across the length and breadth the country called ‘Breathe Easy’ groups. The first group was set up in Birmingham in 1991 [(20), p. 119]. Generally, social support groups bring together people with similar experiences, such as health problems, addictions, disabilities, and particular life circumstances [e.g., (45–50)]. The more formal definition provided by the early pioneers Katz and Bender (51) is that self-help support groups:

…are voluntary, small group structures for mutual aid and the accomplishment of a special purpose. They are usually formed by peers who have come together for mutual assistance in satisfying a common need, overcoming a common handicap or life-disrupting problem, and bringing about desired social and/or personal change. The initiators and members of such groups perceive that their needs are not, or cannot be, met by or through existing social institutions (p. 9).

Given the high importance of social support and psychological capital for quality of life in living with chronic disease (30), social support groups (like Breathe Easy) appear to be a good fit for patients living with COPD. Specifically, support groups seem to result in some various positive outcomes for COPD patients themselves and often for their partners and families as well.

Ethnographically speaking, social interaction within Breathe Easy groups can be understood as a negotiation of various relationships that offers each individual with a ‘safe place’ for making sense of what it is they are saying and doing (52). Drawing from both interviews and fieldwork observations, the author came to identify three primary aspects of what it means to be a Breathe Easy group. These are (1) sociality as support; (2) support as sociality; and (3) advocacy. Firstly, ‘sociality as support’ is described. In brief, this concept is meant to convey that the groups offer a social network in which the aforementioned social condemnations and stigma do not play (a major) part. As such, groups can serve as safe spaces that can serve a therapeutic function as people share emotions and find recovery from stress (53). Friendships are made and social activities are carried out that can prevent people from becoming socially isolated. Moreover, people can exchange mutual help. Yet, for some, this is not seen as ‘support’—as for example one research participant said: ‘But I think for some people it is seen as a support group, but I would not… I would guess… the majority see it more as a social group’. Another research participant even explicitly experienced this as disappointment:

My idea of a self-help group would be people who sit around, having a cup of coffee, and say: “Have you tried this? Have you tried that? Ask your GP about this…” and things like that—but they do not. It’s just a gossip shop. Which is not for me, I’m not a gossip person. That’s all.

This brings us to the second aspect, which is ‘support as sociality’. This is meant to convey the process of acquiring more specific knowledge and skills (‘take-home messages’) on how to deal with a respiratory disease and its symptoms. This process takes place through the social setting that is provided. Or, as one research participant explained it very well:

Yes…… I do find it a support group. If someone has a problem, and they are talking about it, there’s always someone who has had a similar problem. And advise them on that. And, as I said, when we get somebody in from the hospital, and… they are telling them—telling people—well, “There’s aids available… You can get this, and that”—that’s supporting you! And… No, we do [support each other], we get people in who explain about… how your lungs work, how this happens, and… everything. And you just get so much more information, which helps you to cope with it. I think it’s excellent… I’m glad I joined.

Thirdly, social support groups also offer possibilities for advocacy. When it comes to Breathe Easy, some groups (or selected individuals within the groups) engage in fundraising, awareness raising, and lobbying for health service development. Veritably, COPD is still a disease that remains relatively unknown among the public (22). As discussed, for many people it takes a long time before they receive the (correct) diagnosis and can thus access the health services they require. As one research participant said: ‘Plus, I also think that with the fundraising and everything, it’s helping…… our community. The hospital. You know, the people who are involved with COPD, or other breathing problems. It’s helping them’. This seems to be a doubled-edged sword as advocacy can help others, while also providing a good feeling and life quality for the people who are participating in the actual lobbying and advocacy-related activities.

Yet, some people also mention disadvantages that relate to the overall functions of the Breathe Easy groups. For example, people with whom a bond has been built up may pass away, or no longer participate due to deteriorating health. As a partner of one COPD patient said, referring to people who had passed: ‘They were nice, I’m glad I got to know them, you know… But… They’re a big miss, as well’. While the topic of ‘death’ did not stand as a research aim in the ethnographic study (20), it did feature thematically in personal stories and in people’s memories (such as in the former quote). Being that COPD is an incurable, progressive disease, death tends to occur after a ‘prolonged functional decline associated with uncontrolled symptoms, emotional distress and social isolation’ [(54), p. 11]. However, a problem is the increasing evidence that the ‘end of life needs of those with advanced COPD are not being met by existing services’ [(54), p. 11; (55)]. As such, the notion of a ‘good death’ remains more a hope than standard medical practice for all patients involved (55–57). Self-help groups and allied movements can (and do) act as support here—socially, therapeutically, but also through advocacy and health lobbying. Needless to say, patients with severe COPD have a chaotic trajectory towards death and previous research has ‘focused on identifying a “transition point” that would allow identification of those patients who may benefit from a palliative approach to their care’ [(58), p. 310], or referral to specialist palliative care services. Even though persons with COPD are aware of their own mortality, many see their death as distant; therefore, end-of-life wishes are seldomly discussed. Landers and colleagues (58) suggest a new model for the palliative care of patients with COPD where the ‘patient is firmly in the center, surrounded first by their family and then by primary care’ (p. 314). As such, social support (in all its forms) always remains in the limelight. Yet it is also possible that one of these three named functions within the group is present to an extent that is considered unpleasant or impossible. For example, when social activities are organized, it may be that someone’s condition is too poor to participate. As one research participant explained:

But, like, they had a walk a couple of weeks ago—and I knew I could not do it. And I said to one of the ladies I’d met here, “I might come along to support you, and sit on a bench—but I cannot do the walk.” I knew I would not be able to go. Not very far……

As already explained, it may also be that someone mainly needs support as sociality—but finds sociality as support instead. Whereas there may be individual differences in how the presence of dissimilar functions is experienced, it is also true that different support groups may provide a different experience. As Adamsen (59) writes:

There is no clear consensus about the definition and the conceptualization of self-help groups. Terms like self-help groups, support groups, counselling groups, therapy groups and instruction groups are applied randomly both in research and in the clinical practice and this creates uncertainty about whether group initiatives are comparable or not (p. 228).

Given the different preferences found in the current study, however, this is not necessarily a bad thing. After all, this means that there is still room to tailor social support groups to the needs of specific groups and individuals; something that might not just be relevant for COPD support groups. While studies of other types of social support groups have identified similar functions (59–61), no previous attention has been paid to individual preferences in the extent to which these functions are manifested in the groups. Yet, research by Ravenek and Schneider (62) did show that age, and associated stage of Parkinson’s disease, influenced the extent to which patients felt they belonged in a social support group. This parallels with studies that illustrate how participants’ motivations for attending group meetings may change as they move through the treatment trajectory (45). Thus, for patients who are younger than the other group members, it could be a reason for not continuing to participate. Such findings help clarify that people need to sense that what the group has to offer matches who they are and what they are looking for (as individuals).

## 4. Discussion

### 4.1. Limitations and possibilities

Due to its insidious onset, one can conclude that COPD (epidemiologically speaking) is a disease that mainly affects a certain group of people: older adults, and the disease becomes increasingly common with age. Where people are living with COPD, there are a number of clear barriers and risks through which they can experience a lack of social support and psychological capital, which in turn leads to a high treatment burden and reduces their quality of life. This article, which is based on a larger ethnographic study (20), shows that social support groups can play an important role in preventing these issues and the research participants described are predominantly positive about their various experiences. Exceptions to this are mainly found in the mismatch between what each individual group offers, and the needs held by its various members. However, there is no ideal self-help support group. Nor is there ‘a “magical formula” for attracting and retaining a diverse audience’ [(45), p. 447]. Self-help support groups are not to everyone’s liking—especially as ‘not all individuals might benefit equally from support group participation’ [(71), p. 31]. Whereas formats of support groups vary, Breathe Easy groups have always operated through face-to-face meetings. As such, this article does not consider online support groups. Being a physical group in principal does not come without its issues, however. Given that reduced pulmonary function often leads to limitations in mobility (making even the most trivial of tasks physically challenging), Breathe Easy groups usually hold their meetings in venues located in the midst of their respective communities. These venues can be understood as third places (67); that is, environments that are characterized as being neither home (first place) nor work (second place). Dolley and Bosman (67) argue that third places have grown to be especially significant in postmodern times. More specifically, third places offer space for interaction that promotes social attachment and ‘togetherness’ [(67), p. 1] between people in a globalized world characterized by constant mobility and rapid change.

The distinction between offline and online groups may, however, speak to the reason why Breathe Easy groups are primarily attended by older people (65+) and lack attendance from younger persons (despite there not being any age restrictions in place). Namely, ‘[o]nline peer-to-peer communication is popular among young people’ [(63), p. 1]. Yet simultaneously (and perhaps paradoxically), there have been few systematic reviews examining the effectiveness of online peer-to-peer support in improving the health of adolescents and young adults. Criticizing this historical trend, Ali and colleagues write that there is an urgent need to ‘determine the effectiveness of peer support alone as an active intervention’ [(63), pp. 6–7], considering its growing popularity amongst young persons. Having that said, considering how computers and associated technology have become so central to modern life (even ushering in a new era of mass media), in a society where the population is ageing rapidly.

…the acceptance and utilization of developing technologies by an older population is becoming increasingly important […] leading to the conclusion that similar factors influence both [old and young people]—hence, older people could well be taught to use technology in a similar manner to younger people [(65), p. 473].

This may suggest that, sooner or later, older persons around the world may very well turn to using online tools and technologies more often as active health management interventions in their daily lives. However, more research is needed in order to properly map out such a trend, alongside whether online peer support as a sole active health intervention is sufficient to improve the lives of people with different chronic diseases (regardless of their age). However, there may also be pathological reasons as to why online support groups are more prevalent amongst certain populations. For instance, due to the nature of CF (cystic fibrosis), persons living with this disease cannot (and are actively discouraged from) meeting face-to-face (39, 86). Specifically, persons with CF are vulnerable to different bacteria which grow in their lungs. While these bacteria are usually harmless to those who do not have the disease, they can settle in the lungs and be harmful to those people who do (86). Not unexpectedly, the limelight falls once more on the digital divide between the older and younger population, especially when it comes to the increased reliance on digital technologies to deliver healthcare services. As such, the United Nations has called for increased digital inclusion and equality for all (85), where in Europe specifically, only one in four older people have basic or above basic digital skills, compared to ‘two in three in the age group 35–44; three in four among 25–34-year-olds; and four in five among youth (16–24)’ (23). The digital turn in health care notwithstanding, in terms of accessing essential and life-sustaining services the younger population is hugely advantaged whereas older persons are falling behind, needless to say. Digital exclusion is an ongoing global issue (69, 78, 94); an issue only further fueled by the COVID pandemic, with older persons not only being a population extra susceptible to the virus but also to digital exclusion, ‘because they are less likely than younger people to take advantage of the modern information and communication technologies such as smartphones and tablets’ [(94), p. 125]. This extends to COPD treatment regimens and self-management programs as well. Namely, a focus of COPD care is ‘to encourage self-management, particularly during COVID-19, where much face-to-face care has been reduced or ceased’ [(92), p. 1]. The digital turn in health care is, intrinsically, seen to ‘offer affordable and scalable solutions to support COPD patient education and self-management (…)’ where such ‘…solutions could improve clinical outcomes and expand service reach for limited additional cost’ [(92), p. 1]. Nevertheless, in the global endeavor toward ‘digital equality’ the focus remains on the patient—or rather, what the (older) patient lacks (in terms of digital skills) instead of what actually fuels care access disparities. Yet, that is also precisely why self-help support groups exist—for persons with COPD and other chronic conditions: as a public response to (bio)political failures in responding to biomedical misconducts, especially when perceived needs are not (or cannot be) met by or through existing social institutions [cf. (51)]. The lack of access to digital (health) services falls under such misconducts. When all else fails, one turns to mutual aid and other experts-by-experience.

### 4.2. Implications for policy, practice, and future research

With the COVID-19 pandemic, however, amid widespread recommendations to practice social (physical) distancing, the possibilities for arranging face-to-face meetings either vanished completely or were highly restricted. The former was true for persons in the United Kingdom, where a legally enforced ‘Stay at Home Order’ (lockdown) was introduced on March 23rd— banning all non-essential travel and contact with other people (68). Due to these restrictions and considering that COPD patients develop more severe COVID-19 (39), Breathe Easy groups across the United Kingdom were quick to cancel all their face-to-face meetings. While the selected groups which the author met with ultimately turned to partially using digital technologies for their following meetings (now virtual), originally these groups had met in person, and they later went back to arranging face-to-face meetings once the incidence of COVID-19 had decreased and restrictions been lifted. At a population level, mental health during the COVID-19 pandemic was negatively impacted all over the world (81, 93). Yet paradoxically (perhaps), while it was predicted that due to the ‘specific isolation rules for older adults and their heightened risk from the virus, psychosocial consequences such as loneliness would be exacerbated in older age groups’ [(81), p. 1], evidence suggests that older adults, on average, ‘experienced more stable and less negative outcomes compared with other subgroups’ [(81), p. 1]. It goes beyond the scope of this article to explain why this might be the case, but the circumstances were enigmatic to say the least.

Barlow and colleagues once wrote that self-help support groups ‘may rival all other forms of treatment sometime within the next century’ [(64), p. 53]. Only two decades later, support groups are more widespread and multifaceted than ever and are shown to be especially beneficial to improving a person’s skills in self-management (89). Considering that members need to sense that what the group offers matches what they are looking for individually, for policy purposes, this means that improvements can possibly be achieved by looking at this (dis)connection more closely. One option could be to make the social activities and peer-to-peer meetings more diverse to ensure that at least all three primary aspects (i.e., sociality as support; support as sociality; advocacy) are offered to a sufficient extent. Another alternative could be to identify people’s needs early on and direct them to groups that best fit these needs. For example, those people who are mainly interested in support as sociality may prefer support groups that, for instance, have strong connections with and frequently invite healthcare professionals to their meetings. Whereas those individuals who prefer sociality as support, peer-to-peer groups that focus more directly on social activities (e.g., group talks or parlor games) may be more appropriate. All in all, translating the present results into advice for support groups more generally, they point to the importance of better tailoring support groups to the individual in terms of needs and factors that will give a sense of belonging. With respect to the latter, it could well be, for example, that the experiences of same-aged peers are more similar, and that people therefore benefit more from age-homogeneous groups. This would certainly speak to the nature of the Breathe Easy group network, which rarely draws the attention of the younger population. Being that Breathe Easy groups overall are partially autonomous (yet inherently diverse), every group is very much organized so as to fit the needs of its members. Thus, changes to groups’ form and function take place internally, and individually. While each group is set up through funding from the BLF, groups are supposed to retain and practice certain autonomy from the start; especially in terms of how members organize and administer their continuous activities and finances. The same can be said about similarly natured systems of groups; i.e., peer-to-peer and semi-professionally organized support groups, where implications for policy are found by looking at the internal systems of governance. This stands in contrast with professionally operated groups (i.e., clinical and various hospital-based groups), where routines align more closely with established medical practice and legislation (83). As such, the point of issue is what legislators potentially can learn from groups like Breathe Easy and how this knowledge can further inform policy making. Qualitative research, like ethnography, can play a critical role in helping policy makers reach important and well-informed decisions, as well as in identifying those populations who are in most need; would be most receptive; or whose acceptance would ensure broader dissemination of policy development. This article acts as an example of this.

That said, neoliberal reforms have led to deep changes in healthcare systems around the world; especially ‘on account of their emphasis on free market rather than the right to health’ [(87), p. 1]. In the United Kingdom specifically, neoliberal policy has resulted in the propagation of ‘private-capitalist ownership combined with the privatization and commodification of public goods, the public economy and public services’ [(70), p. 168]. People with various disabilities may, in particular, be disadvantaged by such reforms, due to their increased health care needs and lower socioeconomic status (66, 87). Consequently, as respiratory care becomes more individualized (76, 90), this very much affects persons living with COPD and similar conditions. Even more so if we consider how in the United Kingdom, respiratory disease continues to be a major factor in health inequalities (15, 22). As such, the author contends that the increasing admiration for support groups in the United Kingdom may be understood as a direct result of the shifting (neoliberal) responsibilities in the nation’s health care and welfare practices. This is especially evident within respiratory care, which is becoming more and more individualized and now demands new forms of autonomy and patient activation (77).

This is where patient advocacy turns important, which comes to revolve around empowerment and protecting personal autonomy ‘where patients representing themselves benefit from enhanced autonomy and informed consent’ [(88), p. 39]. For Breathe Easy groups, this process may entail fundraising, awareness raising, and lobbying for health service development. However, what Breathe Easy groups ultimately can do with raised funds is very limited. Firstly, the groups are meant to be self-sustained. Thus, groups are allowed to keep whatever they need to cover their monthly costs and remain active. The rest, however, has to be split between the BLF and whatever else the groups would like to fund (i.e., local health care services). As one research participant said:

There’s been a change in the rules… and I think that’s to do with the charity’s guidelines (…) It’s like to do with… the BLF kind of brought them out… these new financial guidelines, but I do not know whether they have been brought out by the charity’s… committee, or trustees (…) like nationally, for all charities or just the BLF; I do not know.

In truth, respiratory disease accounts for over 700,000 hospital admissions and over 6.1 million hospital bed days each year in the United Kingdom [(15), p. 4]. As such, the impact respiratory disease has on health services in the country is comparable to that of non-respiratory cancer or cardiovascular disease. Yet despite this, respiratory disease ‘has not received a similar level of attention and investment, and mortality has stagnated’ [(22), p. 5]. With their impact on health services being on equivalent levels, there is no sound reason for respiratory disease to not be on an equal footing with cancer and cardiovascular disease apropos research funding and public health priorities. Needless to say, research into respiratory diseases continues to be consistently underfunded and the United Kingdom government is not putting a focus on respiratory research, as it has for cancers and cardiovascular disease. Whilst the physiology of breathing is well recognized, the subjective experience of breathlessness is poorly understood. Although far from being an invisible condition (epidemiologically speaking), respiratory health is rarely discussed as the widespread public health issue that it really is (in the United Kingdom and worldwide). Thus, as a result, people living with respiratory diseases often feel invisible and seldom have the energy or confidence with which to challenge those in authority or campaign for improvements in health care.

That said, with self-help support groups in mind, as this article clearly illustrates further research is needed in order to better understand the motivations underlying support group participation. The same goes for self-help culture in general, including various self-improvement products and their impact on society (74, 80). As a health condition, COPD has not remained unchanged as well, and epidemiologically speaking a lot more information is needed in order for treatment regimens to develop further. While the large majority of all cases of COPD are due to tobacco smoke, there are also genetic components to the disease. As such, research into the genetic determinants of severe, early-onset COPD (e.g., alpha-1-antitrypsin deficiency) is needed. Moreover, considering how environmental air pollution is a major determinant of childhood asthma (84), further research is needed on how environmental inequality impacts on respiratory health across all ages (COPD included); especially with regards to ‘unequal distribution of the risks and benefits that stem from interactions with our environment’ [(84), p. 1]. At heart of all these points of issue are, however, the notions of chronic living and the burden of disease, where an untidy mass of string (questions) still stands untangled. Manderson and Wahlberg write (79) that as COVID-19 continues to spread around the world we are, once again.

…reminded of the tenuous epidemiological split between communicable and non-communicable conditions. The latter have long co-occurred with infectious disease, but the mix of the two has typically been illustrated in low and middle-income settings seen to be struggling with a “double burden” of disease, as distinct from high-income settings seen to have entered “the age of degenerative and man-made diseases”… (p. 428).

Ethnographic research stands as an important tool in helping to unravel such complicated subjects; especially as its value lies in its ability to present perspectives from which the world can be apprehended anew. Namely, ethnographic glimpses into the everyday lives of persons living with various chronic and long-lasting conditions (from different, i.e., socio-economic and cultural settings) provide important insights into the ‘very conditions of possibility of chronic living’ (91). Such insights have severe implications for both policy and practice, which cannot be left unheeded.

## 5. Conclusion

Since data collection ended, the lay of the land has changed extensively. The British Lung Foundation (BLF) has now merged with Asthma UK, and they run under a new name: Asthma and Lung UK. Breathe Easy groups still run under this organization, but under a new brand: A + LUK Support Groups. In the transition, already existing groups were given three choices: (1) to remain fully integrated groups; (2) become semi-independent affiliated groups; (3) or become fully independent groups. Many groups picked the second option, and now run as ‘Breathe Easy groups affiliated to A + LUK’. As to avoid confusion in writing in past tense, this article has adhered to the practice of ‘ethnographic present’ (73). That is, the article looks at the engagement with doing fieldwork and writing ethnography “as an ongoing and reflexive process” [(72), p. 1]. As such, Breathe Easy groups are referred to as nothing but ‘Breathe Easy’ groups.

Based on what has been discussed in this article (primarily drawing from ethnographic observations and interviews with several stakeholders), we can conclude that COPD is a disease condition that benefits from gerontological viewpoints—especially considering its correlation with old age and increased longevity. As such, COPD in older persons presents a diagnostic challenge. Namely, the first vital symptom of COPD is breathlessness (29), but as a decline in pulmonary function is also a sign of primary ageing (75) and thus an effect of normal anatomical and physiological modifications, the disease is often masked by an insidious onset understood as a result of ageing. While a cure is not possible, people struggle greatly from the symptoms and need different ways to cope with them. Social support can be very helpful in this, and social support groups overall offer many such possibilities. The results presented are also generalizable and relevant for other chronic diseases, and more specifically those experienced by people in later life. After all, ageing is ‘often associated with multiple long-term health problems influencing older persons’ well-being in daily living’ [(21), p. 1]. Reduced pulmonary function is a symptom of both primary and secondary ageing (75), and often leads to limitations in mobility which makes even the most trivial of tasks physically challenging. This is especially true for COPD and similar conditions, and as such, for many older persons their social network becomes limited. Scholars describe this as experiencing a ‘shrinking’ or ‘diminishing’ lifeworld (21, 41); how the loss of physical capabilities in older persons ‘diminishes the predictability and automatic nature of their bodies and their perceived effectiveness as a person’ [(41), p. 605]. The importance of strong social networks has long been shown to be associated with quality of life for people in later life, especially as it ‘enables mutual practical, emotional and physical support, which can reduce stress…and even illness’ [(82), p. 29]. This article is a clear example of how social support groups can potentially broaden an older adult’s lifeworld and social network by providing a ‘safe space’ where people can feel accepted and exchange similar experiences with one another.

## Data availability statement

The original contributions presented in the study are included in the article/supplementary material, further inquiries can be directed to the corresponding author/s.

## Ethics statement

The studies involving humans were approved by the Department of Anthropology at Durham University, Durham, United Kingdom. The studies were conducted in accordance with the local legislation and institutional requirements. Written informed consent for participation in this study was provided by the participants’ legal guardians/next of kin.

## Author contributions

FN: Conceptualization, Methodology, Writing – original draft, Writing – review & editing.
